# Genetic Mutations That Confer Fluoride Resistance Modify Gene Expression and Virulence Traits of *Streptococcus mutans*

**DOI:** 10.3390/microorganisms9040849

**Published:** 2021-04-15

**Authors:** Hyeon-Jeong Lee, Jihee Song, Jeong Nam Kim

**Affiliations:** 1 Department of Integrated Biological Science, College of Natural Sciences, Pusan National University, Busan 46241, Korea; eyh07@naver.com; 2 Department of Family, Youth, and Community Sciences, University of Florida, Gainesville, FL 32611, USA; ssong@ufl.edu; 3 Department of Microbiology, College of Natural Sciences, Pusan National University, Busan 46241, Korea

**Keywords:** *Streptococcus mutans*, fluoride resistance, phosphoenolpyruvate-dependent phosphotransferase system (PTS), diminished fitness

## Abstract

Fluoride is an inorganic monatomic anion that is widely used as an anti-cariogenic agent for the control of caries development. The aims of this study were to identify the mutated genes that give rise to fluoride-resistant (FR) strains of the cariogenic pathogen *Streptococcus mutans* and explore how genetic alterations in the genome of an *S. mutans* FR strain optimize the metabolism(s) implicated in the expression of virulence-associated traits. Here, we derived an *S. mutans* FR strain from a wild-type UA159 strain by continuous shifts to a medium supplemented with increasing concentrations of fluoride. The FR strain exhibited a slow growth rate and low yield under aerobic and oxidative stress conditions and was highly sensitive to acid stress. Notably, microscopy observation displayed morphological changes in which the FR strain had a slightly shorter cell length. Next, using the sequencing analyses, we found six mutations in the FR genome, which decreased the gene expression of the phosphoenolpyruvate-dependent phosphotransferase system (PTS). Indeed, the ability to intake carbohydrates was relatively reduced in the FR strain. Collectively, our results provide evidence that the genetic mutations in the genome of the FR strain modulate the expression of gene(s) for carbon metabolism(s) and cellular processes, leading to diminished fitness with respect to virulence and persistence.

## 1. Introduction

Dental caries is a common oral disease closely associated with the increasing intake of fermentable carbohydrates due to changes in food processes and dietary habits [1]. Globally, approximately 2.4 billion people have caries on permanent teeth, and the prevalence varies by region and country among individuals with different cultural backgrounds [2]. In the oral cavity, more than 700 species of microorganisms are present, of which *Streptococcus mutans* and *Streptococcus sobrinus* are dominantly isolated in dental caries [3,4,5]. In particular, *S. mutans* is one of the major agents responsible for the initiation of human dental caries, which forms dental plaque (dental biofilm) on the tooth surface. During biofilm development, *S. mutans* induces plaque acidification through the production of various organic acids via fermentation using monosaccharide or disaccharide molecules, amino sugars, and other sugar derivatives along with the accumulation of extracellular polysaccharide matrix. Consequently, the acidic pH within the dental biofilm leads to an increased proportion of acid-tolerant microorganisms and promotes the occurrence and progression of caries [4,6]. 

Although dental caries is a preventable disease, lack of dental care and poor oral hygiene behavior are still considered the main causes of caries. To control pathogen-mediated caries, many oral hygiene products or dental materials composed of chemical compounds, such as chlorhexidine, cetylpyridinium chloride, triclosan, and fluoride, have been applied. Among these chemicals, fluoride is commonly used as an anti-cariogenic agent because it not only exerts an antibacterial effect but also protects the tooth surface [7]. In fact, the personal use of oral hygiene products containing fluoride decreases the prevalence of caries by 24–26% in permanent teeth [8]. Moreover, fluoridation of water in the range of 0.5 to 1.0 mg L^−1^ is a cost-effective method to moderate the potential of cariogenesis [9]. 

In contrast to the benefits of fluoride on oral health, negative effects, such as the emergence of fluoride-resistant (FR) strains, have become concerning. Previous studies have demonstrated this possibility in both clinical (although scarce) and laboratory conditions and analyzed their resistance mechanisms. This acquired resistance can be either transient or permanent through phenotypic adaptation or genotypic modification. Clinical studies with an *S. mutans* FR strain isolated from radiation-treated cancer patients with xerostomia (dry mouth) have suggested that the fluoride resistance is unstable and transient via horizontal gene transfer of plasmid DNAs [10,11]. Further, to better understand the resistance mechanism, other research groups have attempted to create FR strains of *S. mutans* or *Enterobacter cloacae* species under laboratory conditions [12,13,14]. The laboratory-derived FR strains harboring multiple single-nucleotide polymorphisms (SNPs) present stable, permanent resistance. Interestingly, the physiological outcomes among resistant strains from different sources are inconsistent. For instance, the FR strain from *S. mutans* C180-2 had a better ability to form biofilms than the parental strain [15], whereas the FR strain derived from the *S. mutans* 6715 strain showed reduced biofilm production [10]. 

Enolase and ATPase, which are sensitive to fluoride, have been considered as possible enzymes involved in fluoride resistance [16]. Enolase, also named phosphopyruvate hydratase, participates in glycolysis as a cytosolic metalloenzyme responsible for the conversion of 2-phosphoglycerate (2-PG) to phosphoenolpyruvate (PEP). Fluoride is known to inhibit sugar transport through the PEP:carbohydrate phosphotransferase system (PTS) in oral streptococci through its action on enolase [17]. Moreover, fluoride can diminish the acid tolerance of oral streptococci by inhibiting proton translocation through the F_o_ domain of ATPase in the membrane [18,19]. Previous reports have demonstrated that the enolase activity of sensitive and resistant strains is markedly reduced in the presence of sodium fluoride (NaF) under low pH conditions [20,21,22]. However, no, or minor, differences in glucose uptake level and ATPase activity have been observed for the FR strain [16,21]. This phenomenon has been discussed with the possibility that the FR strain may possess malfunctioned enolase by a mutation in the coding region, allowing it to become less sensitive to fluoride [16] or altering glycolysis by activating alternative pathways [22].

Recently, it was discovered that the fluoride riboswitch activates the expression of *crcB* and *eriC^F^* that encode fluoride transporters in *Escherichia coli* and *Pseudomonas syringae*, respectively [23,24]. However, no fluoride riboswitch has been found in *S. mutans* genomes, and this bacterium encodes two *eriC^F^* homologs, *SMU.1289c* (named *permease_B*) and *SMU.1290c* (named *permease_A*). These homologs are also annotated as *eriC1b* and *eriC1a*, respectively [12,25,26]. One research group showed that both *eriC1a* and *eriC1b* are required for growth in a fluoride concentration to which the wild-type strain is susceptible [26]. However, another group has reported controversial results that only the inactivation of the *eriC1b* gene results in severe growth impairment [25]. 

To date, several candidate genes directly or indirectly linked to the FR phenotype have been introduced. In particular, Liao et al. compared the genomes of FR strains derived from *S. mutans* C180-2 and UA159 and identified three common loci with SNPs [22]. These two strains harbor shared mutations in the promoter region of *mut* (encoding putative mutase) and *glpF* (encoding putative glycerol uptake facilitator) and in the coding region of the *pyk* gene (encoding pyruvate kinase) [22]. However, it is unclear how acquired mutations in an *S. mutans* FR strain influence the transcription levels of genes for certain metabolism(s) associated with fluoride resistance. To address this important knowledge gap, we analyzed the genome of a FR strain that was generated in our laboratory and identified mutations that contribute to fluoride resistance. Moreover, phenotypic changes linked to the transcriptional response induced by these genetic mutations were evaluated. Overall, our results reveal that the expression of genes involved in biochemical pathways can be modulated by acquiring the resistant phenotype to optimize the biological fitness of an *S. mutans* FR strain.

## 2. Materials and Methods

### 2.1. Isolation of *Streptococcus mutans* Fluoride-Resistant (FR) Strains

An *S. mutans* FR strain was obtained from a wild-type UA159 strain through continuous shifts to brain heart infusion (BHI) medium (BD Biosciences, New Jersey, NJ, USA) supplemented with increasing concentrations of fluoride, as described previously [14,16] with some modifications. Briefly, an *S. mutans* wild-type strain was grown to the late-exponential phase (optical density at 600 nm wavelength, OD_600_ = 0.6–0.7) in BHI medium at 37 °C in a 5% CO_2_ atmosphere. The culture was inoculated on BHI agar supplemented with 100 μg mL^−1^ of NaF (JUNSEI, Tokyo, Japan) and grown at 37 °C in 5% CO_2_ conditions. Following incubation, a visible colony was again inoculated on fresh BHI agar supplemented with increasing concentrations of NaF per 100 μg mL^−1^ increment. This sub-culturing was repeated until the maximum concentration of NaF reached 1 mg mL^−1^. To confirm the stability of fluoride resistance, the mutant colony, which was grown in a 1 mg mL^−1^ final concentration of NaF, was sub-cultured 20 times through fresh NaF-free BHI agar. Following these passages, the FR cells was able to grow on BHI agar supplemented with 1 mg mL^−1^ NaF.

### 2.2. Growth Measurements

For measuring the growth of *S. mutans* wild-type and FR strains, overnight cultures were diluted 1:100 in fresh BHI medium and anaerobically incubated to early exponential phase (OD_600_ = 0.2–0.3) at 37 °C. These cultures were then diluted 1:100 in fresh BHI medium and grown under aerobic or anaerobic conditions. To generate anaerobic conditions, the diluted cultures were overlaid with sterile mineral oil. To evaluate the sensitivity to acid stress, the pH of the BHI medium was adjusted to 5.5 by adding 6 N HCl solution. For generating oxidative stress conditions, hydrogen peroxide (H_2_O_2_) was added to the medium at concentrations increasing by 0.003%. The optical density of cells growing at 37 °C was measured at a 600 nm wavelength for 24 h at 1-h intervals using a Bioscreen C plate reader (Oy Growth Curves Ab Ltd., Helsinki, Finland).

### 2.3. Visualization of Cell Morphology and Biofilm

Cell morphology was observed using field emission scanning electron microscopy (FE-SEM) [27]. Cells were grown to the late-exponential phase (OD_600_ = 0.6–0.7) in BHI medium at 37 °C in a 5% CO_2_ atmosphere. The cultures were placed on either a slide glass or hydroxyapatite (HA) disc and fixed using 10 μL of 2.5% glutaraldehyde in phosphate buffered saline (PBS) overnight at 4 °C. The samples were washed twice with PBS and dehydrated using increasing concentrations of ethanol (25%, 50%, 75%, 90%, and 100%) for 10 min. Following dehydration, the samples were coated and visualized using a SUPRA25 FE-SEM (Carl Zeiss, Oberkochen, Germany). 

To assess biofilm formation, early exponential cultures grown in BHI medium were diluted 1:100 in a semi-defined biofilm medium (BM) [28] supplemented with 18 mM glucose and 2 mM sucrose as carbon sources. Cells were grown in a Nunc Lab-Tek II Chamber Slide System (Thermo Fisher Scientific, Waltham, MA, USA) at 37 °C in 5% CO_2_ conditions for 24 h. The biofilms were washed twice with distilled water and stained using the LIVE/DEAD BacLight Bacterial Viability Kit (Invitrogen, Carlsbad, CA, USA) for 15 min, as described in the manufacturer’s manual. The prepared biofilm samples were visualized using the LSM-800 confocal laser scanning microscope (CLSM; Carl Zeiss, Oberkochen, Germany).

### 2.4. Genomic DNA Extraction and Whole-Genome Sequencing

*S. mutans* wild-type and FR strains were incubated at 37 °C in 5% CO_2_ conditions for 24 h in BHI medium and harvested by centrifugation at 16,000× *g* for 2 min at 4 °C. Genomic DNA was extracted using GeneElute Bacterial Genomic DNA Kits (Sigma-Aldrich, St. Louis, MO, USA), according to the manufacturer’s instructions. Genomic DNA (1 μg) was sequenced using Illumina HiSeq 2500, and the raw data were analyzed by Theragen (Suwon, South Korea). Briefly, FASTQ files were trimmed using the Sickle tool, filtered, and mapped using the BWA tool. Data were converted to a BAM file by sorting, merging, and de-duplication using the Picard tool. The BAM files were converted to a variant call format that informs genetic variations using the GATK software. Variant annotation was performed using SnpEff. Following statistical analysis, the output file was analyzed using the Genome Workbench program in the NCBI database (https://www.ncbi.nlm.nih.gov/tools/gbench/, accessed on 11 March 2021).

### 2.5. RNA Extraction and Transcriptome Analysis 

Cells were grown to the late-exponential phase (OD_600_ = 0.6–0.7) in BHI medium at 37 °C in a 5% CO_2_ atmosphere. The cells were harvested and stabilized with 1 mL of RNAprotect Bacteria Reagent (Qiagen, Hilden, Germany). The cells were then pelleted by centrifugation at 18,000× *g* for 10 min at 4 °C and resuspended in 50/10 mM Tris-EDTA buffer with sodium dodecyl sulfate. The resuspended cells were transferred to a 2-mL screw cap tube containing acid phenol and 0.1 mm glass beads and disrupted using a Bead Beater (Biospec Products, Inc., Bartlesville, OK, USA). Total RNA was extracted using a RNeasy Mini Kit (Qiagen, Hilden, Germany) according to the manufacturer’s instructions. The concentration of RNA was determined using a NanoDrop 2000 Spectrophotometer (Thermo Fisher Scientific, Waltham, MA, USA). 

One microgram of total RNA was used to construct the cDNA library. Paired-end sequencing of the constructed cDNA libraries was performed using Illumina Hiseq 4000 (Macrogen, Seoul, South Korea). Raw data from the RNA-seq were mapped to a reference genome (*S. mutans* UA159) using Galaxy online (https://usegalaxy.org, accessed on 11 March 2021). FASTQ files were converted to format using the FASTQ groomer tool and confirmed through the FastQC tool. Output files from the FASTQ groomer were aligned with a reference genome of *S. mutans* UA159 using Bowtie. Mapped reads were converted to BAM files, and the transcripts were assembled and quantified using StringTie. Cufflink assemblies were merged together by Cuffmerge. Quartile library normalization and pooled dispersion estimation methods were performed for differential expression analysis. The functional annotation of the filtered data was performed using the Database for Annotation, Visualization, and Integrated Discovery (DAVID) program (https://david.ncifcrf.gov, accessed on 11 March 2021). Based on the results of functional annotation, the Kyoto Encyclopedia of Genes and Genomes (KEGG) mapper displayed a major pathway that includes a significant difference in gene expression between wild-type and FR *S. mutans* strains. 

### 2.6. Quantitative Real-Time Reverse Transcription-Polymerase Chain Reaction (qRT-PCR)

qRT-PCR was performed to quantify the expression levels of target genes. cDNA was synthesized using the PrimeScript RT reagent kit (Takara, Shiga, Japan) and random hexamer from 1 μg of total RNA. qRT-PCR was performed using a StepOnePlus Real-Time PCR system (Applied Biosystems, California, USA) using 2 × qPCR master mix (with EvaGreen, high ROX) (Coregen, Busan, Korea) and gene-specific primers (Table S1). The qRT-PCR conditions were as follows: initial denaturation step for 15 s at 95 °C, followed by 40 cycles of denaturation for 20 s at 95 °C, annealing for 30 s at 50 °C, and extension for 30 s at 72 °C. All reactions were performed in triplicate, and the expression levels were normalized to those of the 16S rRNA gene as an endogenous control. Fold changes in gene expression were represented using the 2^−ΔΔCt^ method.

### 2.7. PEP-Dependent PTS Assay

PTS-dependent transport activity was determined as described previously [29,30], with some modifications. Briefly, an overnight culture of *S. mutans* strains was grown in tryptone–vitamin (TV) medium [31] supplemented with 0.5% glucose to mid-exponential phase (OD_600_ = 0.5–0.6) and harvested. Cells were washed twice with 0.1 M sodium potassium phosphate buffer containing 5 mM MgCl_2_ (pH 7.2) and resuspended in 0.1 volume of the same buffer. The resuspended cells were permeabilized using a toluene–acetone (1:9) solution by vortexing twice for 2 min at 2-min intervals. The reaction mixture was prepared by the addition of 0.05 mM NADH, 5 mM NaF, 5U lactate dehydrogenase (LDH), and 50 mM of the desired carbohydrate to the permeabilized cells. The reaction was initiated by the addition of 2.5 mM PEP, and the rate of PEP-dependent oxidation of NADH was monitored at a 340 nm wavelength using a Multiskan FC microplate reader (Thermo Fisher Scientific, Waltham, MA, USA). The activity values were normalized to the concentration of total protein in the permeabilized cells determined with a bicinchoninic acid assay (Thermo Fisher Scientific, Waltham, MA, USA).

### 2.8. pH Drop Assay

Acid production in *S. mutans* strains was measured as described previously [32]. Mid-exponential cultures (OD_600_ = 0.5–0.6) of *S. mutans* wild-type and FR strains grown in BHI medium were harvested for 10 min at 4 °C. The cells were washed twice with cold water and resuspended in 4.75 mL of 50 mM KCl with 5 mM MgCl_2_ solution. The pH of the reaction mixture was adjusted to 7.2 by the addition of 0.1 M KOH solution, and 50 mM (final concentration) of the appropriate carbohydrate was added to initiate the assay. The pH change was monitored at 1-min intervals for 30 min using the SevenCompact pH meter S220 (Mettler-Toledo, Columbus, OH, USA). 

### 2.9. Statistical Analysis

All experiments were repeated at least in triplicate with three individual cultures. For statistically comparing the data, one-way analysis of variance was performed. In all cases, *p* < 0.05 was considered significant in most assays performed, and RNA-seq results were considered significant at *p* < 0.01.

## 3. Results

### 3.1. Isolation of a *Streptococcus mutans* Fluoride-Resistant (FR) Strain

**Figure 1:**
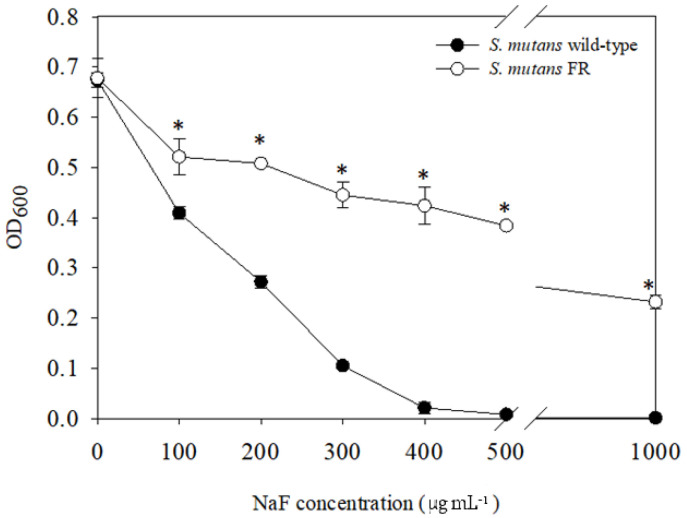
Growth of *S. mutans* wild-type and FR strains in the presence of fluoride. Cells were grown in BHI medium supplemented with increasing concentrations of NaF at 37 °C in a 5% CO_2_ atmosphere for 24 h. The end-point growth was determined according to the OD_600_ value using the Bioscreen C Lab System. The results represent the mean values of three biological repeats. *, differs from the wild-type strain at *p* < 0.05.

As described above, the phenotypes of the FR strains created by different groups appeared to be inconsistent [14,16]. Considering the distinct characteristics of these resistant strains isolated from different experimental environments, we attempted to generate a new FR strain of *S. mutans* using the procedure described in the Methods section. To determine the stability of fluoride resistance, we determined the end-point growth in BHI medium containing serial concentrations of NaF. As shown, the growth of an FR strain was observed in up to 1 mg mL^−1^ of NaF, whereas no growth of the UA159 strain was observed at concentrations higher than 400 µg mL^−1^ (*p* < 0.05; Figure 1). Thus, the *S. mutans* FR strain exhibited higher fluoride resistance than the parental strain. Moreover, confirmation that the laboratory-derived FR strain acquired stable, permanent fluoride resistance was made through 20 passages in fluoride-free medium.

### 3.2. Genomic Analysis Revealed Six Genetic Mutations in the FR Strain 

Previous studies have identified genes that contribute to fluoride resistance in different species, and several gene candidates have been found in bacteria such as oral streptococci and *Enterobacter cloacae* FRM [13,25]. Genome sequencing was performed to identify mutation(s) contributing to the resistance phenotype of the FR strain. As shown in Table 1, the FR strain harbored six mutations in the genome compared to the reference genome of *S. mutans* UA159 (Reference No, NC_004350.2).

The genomic DNA of the FR strain appeared to include a multiple-nucleotide insertion (563th A to “ACAGAATTGACCT”) in the *hsdS* gene that encodes a putative restriction endonuclease, single nucleotide substitutions in the coding region of four genes (*glpF*, C464A; *pykF*, G1156A; *murC2*, G289T; and *SMU. 2059c*, A707T), and single nucleotide substitutions found in the intergenic region between two genes (*SMU.1289c* and *SMU.1290c*, A to G), when compared to the laboratory stock UA159 wild-type genome (Table 1). The *glpF* gene, encoding a glycerol uptake facilitator protein, of the FR strain contained a nonsense mutation that led to the generation of a stop codon as the sequence “C” was changed to “A” at the 464th nucleotide. The *pykF* gene is a putative pyruvate kinase that is an essential gene for glycolysis, converting PEP to pyruvate. In the FR strain, a *pykF* gene harbored a missense variant in which the 1156th guanine was mutated to adenine, leading to an amino acid change from valine to isoleucine. The *murC2* gene, which encodes a putative UDP-N-acetylmuramyl tripeptide synthetase, had a missense variant in which the 289th nucleotide guanine was changed to thymine, resulting in a change in amino acid from alanine to serine. The 707th adenine in the *SMU.2059c* gene, encoding a putative integral membrane protein, was changed to thymine, leading to a change from tyrosine to phenylalanine. Interestingly, the intergenic regions of these two genes, *SMU.1289c* and *SMU.1290c*, which encode chloride channel permeases, contained a mutation in which adenine was changed to guanine. Importantly, among these mutated genes, there is previous evidence that *glpF, pykF, SMU.1289c*, and *SMU.1290c* are potentially major contributors to the fluoride resistance of the *S. mutans* strain [12,22], although the mutation positions were not identical.

### 3.3. FR Strain Shows Impaired Growth and Low Stress Tolerance

**Figure 2:**
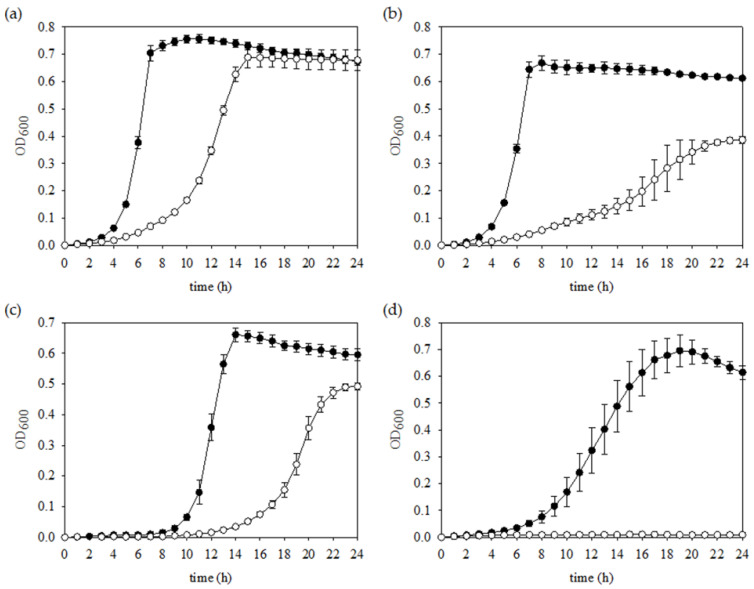
Growth phenotypes of *S. mutans* wild-type and FR strains under various stress conditions. Cells were grown in triplicate to mid-exponential phase in BHI medium and diluted 1:100 into fresh BHI medium (**a**) with a mineral oil overlay, (**b**) without a mineral oil overlay, (**c**) supplemented with 0.003% H_2_O_2_, or (**d**) supplemented with HCl to lower the pH to 5.5 in anaerobic conditions. The symbols (closed and open circles) indicate *S. mutans* wild-type and FR strains, respectively.

To assess the growth characteristics of the FR strain, we compared the growth of the wild-type and FR strains under aerobic or anaerobic conditions. While no significant difference in the final yield was found, the anaerobic growth of the FR strain showed slower growth rates (doubling time, 156.54 ± 4.07 min) when compared to the parental strain (53.78 ± 1.90 min) (*p* < 0.05) (Figure 2a). Similarly, when cells were aerobically grown in plain BHI broth, the FR strain exhibited an apparent growth defect (399.16 ± 62.04 min) along with lower growth of population than the wild-type strain (58.65 ± 0.84 min) (*p* < 0.05) (Figure 2b). 

*S. mutans* is susceptible to oxidative stresses caused by H_2_O_2_ that generates primarily hydroxyl-radical species, which are produced by peroxigenic bacteria to compete with *S. mutans* [33,34]. When treatment with H_2_O_2_ was applied, a slower growth rate and greatly extended lag phase of the FR strain were measured than those shown for the wild-type strain (Figure 2c), indicating that the intact products from the genes mutated in the FR strain are needed for optimal exponential growth in oxidative stress conditions. Furthermore, acid tolerance is recognized as essential for the cariogenic potential of *S. mutans* [34]. Unexpectedly, no growth of the FR strain was observed, whereas the wild-type strain grew well under the same conditions (Figure 2d). Therefore, the FR strain had impaired growth properties regardless of oxygen pressure and significantly lowered tolerance to an acidic stressor.

### 3.4. Morphological Change of the FR Strain

**Figure 3:**
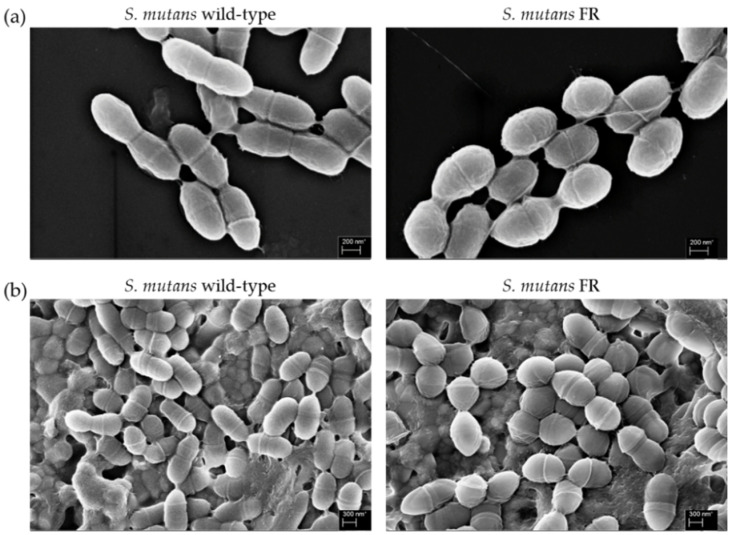
Cell morphology. The wild-type and FR *S. mutans* strains were grown to late-exponential phase (OD_600_ = 0.6–0.7) in BHI medium at 37 °C in a 5% CO_2_ atmosphere. The cultures were fixed on either (**a**) a glass slide or (**b**) HA disc and observed at 30,000× magnification using a SUPRA25 FE-SEM.

According to the bacterial growth law, growth rate, and cell size are known to have a positive relationship with nutrient availability, accompanied by central carbon metabolism [35]. Thus, it is possible that the growth defect of the FR strain is linked to cell size or shape. To confirm this hypothesis, cell morphology of the wild-type and FR strains was observed by growing cells in plain BHI and visualizing using FE-SEM (Figure 3). The samples were prepared as detailed in the Methods section and visualized at ×30,000 magnification. As shown in Figure 3a, the typical oval-shaped cells were observed, which was apparent in the UA159 strain. However, the FR sample contained abnormally shaped cells, which were more compact and rounded (Figure 3b). By comparison, the FR strain showed a relatively smaller cell size (595.95 ± 12.58 nm) than the wild-type strain (621.62 ± 11.47 nm) (*p* < 0.05). In addition, the cell surface of the FR strain appeared to be more rough with obscure division septa. To assess cell morphology in biofilms, the culture was incubated on a HA disc, similar to the major component of tooth and observed at ×15,000 magnification. Consistent with the above results, the FR sample displayed the absence of normally shaped cells, which were observed in the wild-type strain (Figure 3B). Thus, these results indicated that multiple mutations in the FR strain affected the coordination of growth rate and cell morphology of *S. mutans*.

### 3.5. FR Strain Lowers the Ability to Form Biofilms

**Figure 4:**
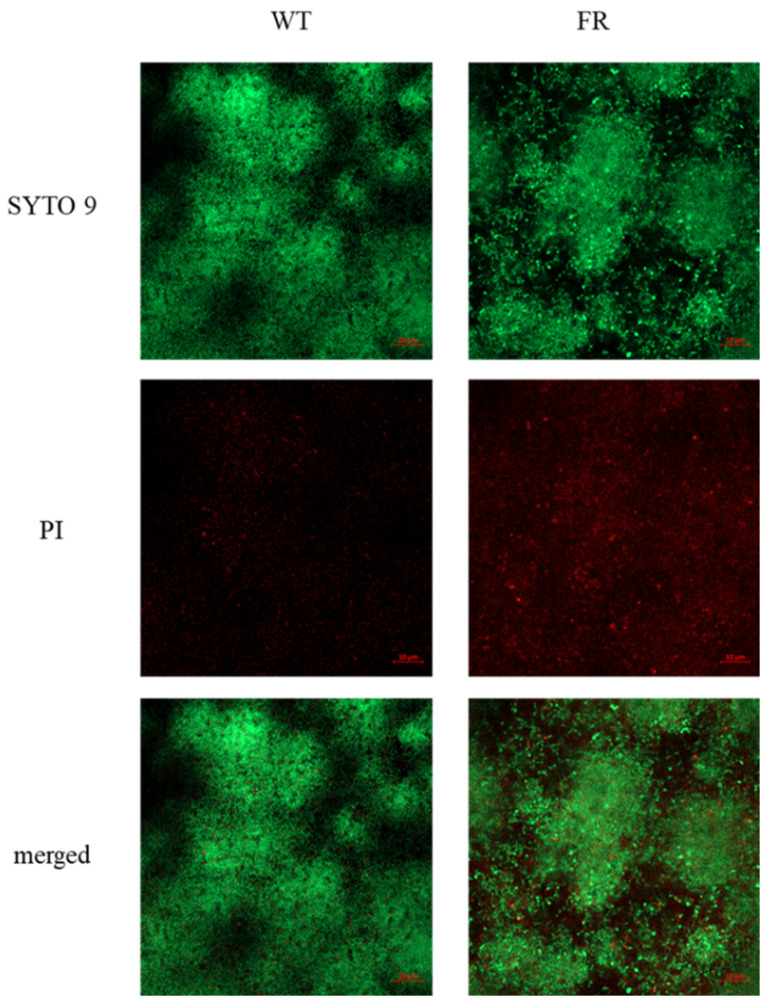
Comparison of biofilm formation between *S. mutans* wild-type and FR strains. *S. mutans* wild-type and FR strains were grown to an OD_600_ value of 0.3 in BHI broth, diluted 1:100 in semi-defined BM supplemented with 18 mM glucose and 2 mM sucrose, and incubated at 37 °C in a 5% CO_2_ atmosphere for 24 h. Biofilms of wild-type and FR strains were observed using the LSM-800 CLSM, after staining using the LIVE/DEAD BacLight Bacterial Viability Kit. Images presented are a representative set of three independent experiments.

Biofilm formation is recognized as an important virulence factor that promotes *S. mutans* adherence to the tooth surface, providing protection or tolerance against external stressors and helping to retain energy sources [36]. According to Cai and colleagues, the FR *S. mutans* UAFR strain that harbors multiple genetic mutations displays reduced fitness in biofilms [15]. However, the engineered UA35 strain harboring a single point mutation in the promoter region of the *mut* gene behaves similar to a wild-type UA159 strain. To evaluate biofilm formation by the FR strain, images of the biofilms were obtained using a CLSM by growing cells in a defined BM after 24 h of incubation (Figure 4). Biofilms of the wild-type strain were densely distributed and covered most of the substratum. In contrast, the FR strain formed relatively thinner biofilms that were sparse and spatially clustered. Particularly, the green fluorescence intensity, corresponding to live cells, in the wild-type biofilms was slightly stronger than that in the FR biofilms, which was similar to the live cell count determined by CFUs (Figure S1). Therefore, these results provide evidence that the aberrant architecture of biofilms formed by the FR strain may be related to the live cell population and its slow growth rather than a reduced ability to form biofilms.

### 3.6. Transcription Profiling of an FR Strain

RNA-seq was performed to assess the differential expression of genes in the FR strain and investigate the relationship between phenotypic variations and transcriptional response by gene mutations that contribute to fluoride resistance in *S. mutans*. Through transcriptome analysis, genes or operons that differed by at least ≥1.0 or ≤ −1.0 log_2_ values in expression level were sorted and designated as upregulated (Table S2) or downregulated genes, respectively (Table S3). Overall, the expression of 106 genes was increased in the FR strain, whereas the expression of 22 genes or operons was reduced (*p* < 0.01). To determine the contribution of fluoride resistance to cellular processes or metabolisms, functional analysis with the RNA-seq data was performed using the DAVID algorithm. Overall, the expression of genes for ABC transporters, PTS, fructose and mannose metabolism, fatty acid metabolism, and fatty acid biosynthesis were significantly influenced in the FR strain. Notably, the expression of malXFGK in the maltose/maltodextrin ABC transporter increased [37,38], and four genes (*msmEFG*, *gtfA*) of the *msm* operon for the metabolism of multiple sugars [39,40] were found upregulated. These two ABC transporters appear to be involved in the uptake of disaccharides and/or oligosaccharides, and the substrate specificity of MalXFGK is distinct from that of MsmEFGK [38]. Additionally, the upregulation of *glgP* and *malQ* genes, annotated as putative glycogen phosphorylase and putative 4-alpha-glucanotransferase, respectively, were observed. These two genes are co-transcribed in an operon, negatively governed by the MalR regulator [41]. In addition, a study of purified MalQ and GlpF proteins has demonstrated that the coordination of glucose-releasing and phosphorylase activities by MalQ and GlpF proteins, respectively, allows *S. mutans* cells to ferment starch-degradation products (e.g., maltose and maltodextrins) in the oral environment [41]. 

In contrast to the upregulated genes for carbohydrate uptake and utilization, the expression of the mannose PTS transport system encoded by *SMU.1961c*, *SMU.1958c*, and *SMU.1957* (annotated *manXYZ*), along with the glucose/mannose enzyme II (EII) permease encoded by *manLMN* genes was markedly downregulated in the FR strain. GlcNAc and GlcN are the most commonly found amino sugars and serve as building blocks for the peptidoglycan of bacterial cell walls and certain lipopolysaccharides [42,43,44]. GlcNAc and GlcN can be internalized and phosphorylated through the *manXYZ*-encoded transporter and then converted to N-acetylglucosamine-6-phosphate (GlcNAc6P) or glucosamine-6-phosphate (GlcN6P), respectively, which serve as important allosteric effectors for downstream metabolic processes [45,46]. Recent studies have shown that these amino sugars are transported through the PTS system, with the permease encoded by *manLMN* playing a dominant role in *S. mutans* [30]. Taken together, the differential expression in various PTS transports coupled with carbohydrate metabolism was mainly detected, which suggests that the FR strain has a change in specificity and affinity to the carbohydrate substrates.

**Figure 5:**
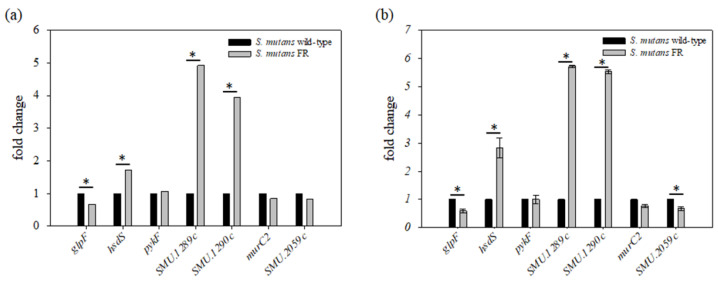
Transcriptional analysis of genes harboring a mutation. (**a**) Transcriptome analysis was performed by RNA-seq technique using total RNA from the wild-type and FR strains, as described in the Materials and Methods. Transcriptome analysis generated a graph that displays the fold-change values of the selected seven genes bearing a mutation in the FR strain compared to that of the wild-type strain; (**b**) Comparison of gene expression between *S. mutans* wild-type and FR strains measured by qRT-PCR. The wild-type and FR strains were grown to late-exponential phase (OD_600_ = 0.6–0.7) in BHI medium at 37 °C in a 5% CO_2_ atmosphere. cDNA was synthesized with a random hexamer from 1 μg of total RNA. The data indicate the fold changes of the target RNA derived from 1 µg of input RNA after normalization to the 16S rRNA levels in the same samples. *, differs from the wild-type genetic background at *p* < 0.05.

Next, to explore the influence of the identified mutations that led to fluoride resistance on gene expression, transcription of the mutated genes and genes bearing a mutation in the intergenic region was evaluated (Figure 5a). We further compared the expression pattern of these genes with the results from the qRT-PCR analysis (Figure 5b). The expression of the *glpF* gene was decreased by 0.5-fold (*p* < 0.05), whereas the expression of *hsdS*, *SMU.1289c*, and *SMU.1290c* genes exhibited significant enhancement (*p* < 0.05). However, no significant difference in the expression of *pykF*, *murC2*, and *SMU.2059c* genes was observed between the wild-type and FR strains. Therefore, the expression of *glpF*, *hsdS*, *SMU.1289c*, and *SMU.1290c* genes was substantially modulated in the FR strain, suggesting that these mutated genes are a primary contributor toward conferring fluoride resistance.

### 3.7. Fluoride Resistance Affects Carbohydrate Uptake

**Figure 6:**
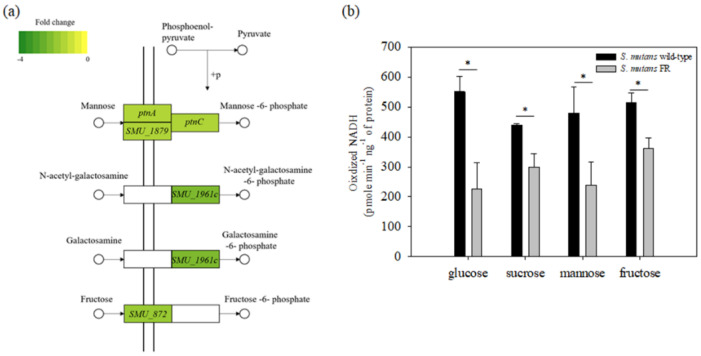
(**a**) KEGG diagram indicating genes that are a part of the PTS. These genes are highlighted in green, with the color intensity corresponding to their level of downregulation; (**b**) PTS transport of glucose, sucrose, mannose, or fructose was determined using permeabilized cells as described in the Materials and Methods. *, differs from the wild-type genetic background at *p* < 0.05. The results are expressed as mean values for three biological repeats, with error bars indicating the standard deviation.

In general, the PTS system is known to catalyze the uptake and concomitant phosphorylation of carbohydrates using PEP as an energy source and phosphoryl donor [30,47,48,49]. As described above, the expression of PTS-specific genes, specifically for mannose-specific PTS, was found to be downregulated in the FR strain (Figure 6a). In contrast, we also found that the major glucose transporters encoded by the *ptsG* gene and multiple sugar transporters, including *malXFGK* and *msm* operon, were upregulated (Table S2). To confirm whether the downregulation of the five genes (*ptnA*, *ptnC*, *SMU.1879*, *SMU.1961c*, and *SMU.872*) affects the ability to uptake sugars in the FR strain, we determined the ability of PTS to uptake carbohydrates, which is represented by the amount of oxidized NADH (Figure 6b). When using glucose, sucrose, mannose, and fructose as sole carbon sources, the amount of oxidized NADH in the wild-type strain was 550.88 ± 49.68, 439.37 ± 4.48, 479.48 ± 86.90, and 515.17 ± 31.48 pmole min^−1^ ng^−1^ of protein, respectively. In the FR cells, the oxidized NADH corresponding to the four tested carbohydrates was 227.19 ± 86.79, 300.06 ± 42.54, 238.53 ± 77.15, and 360.33 ± 36.69 pmole min^−1^ ng^−1^ of protein, respectively. Thus, the FR strain had lower PTS activity than that of the wild-type strain in conditions supplemented with the tested carbohydrates (*p* < 0.05).

### 3.8. Production of Acid-End Products Is Reduced in the FR Strain

**Figure 7:**
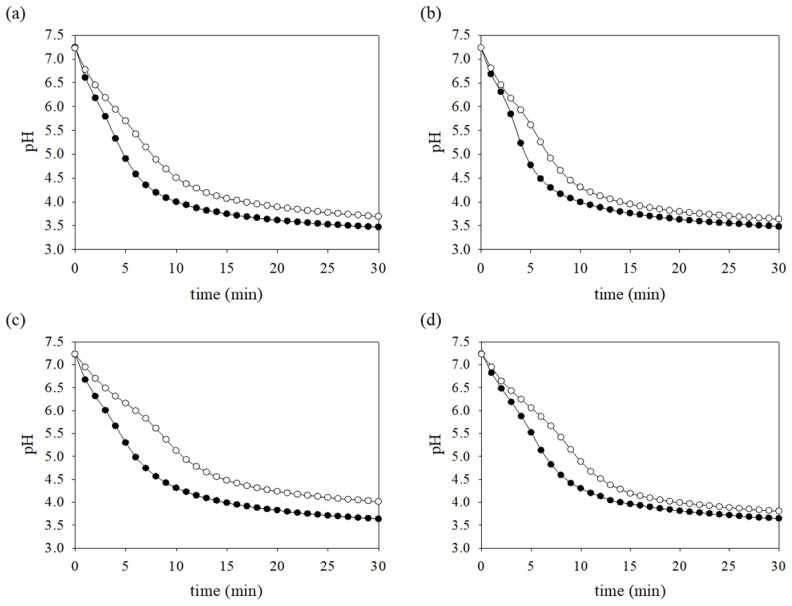
pH drop assays. *S. mutans* wild-type and FR strains were grown to mid-exponential phase in BHI medium supplemented with 20 mM glucose. The cultures were subjected to pH drop assays as described in the Materials and Methods. The assay was initiated by the addition of 50 mM (**a**) glucose, (**b**) sucrose, (**c**) mannose, or (**d**) fructose. The change in the pH was monitored at 1-min intervals for 30 min. The data represent the averages of results of three independent experiments. The symbols (closed and open circles) indicate *S. mutans* wild-type and FR strains, respectively.

*S. mutans* produces organic acids, specifically lactic acid, as an acid-end product via glycolysis, which is an essential virulence factor in the establishment of caries development [48]. In fact, glycolytic flux following carbohydrate intake is critical for the biosynthesis of acid products and ATP generation. To test whether the lower PTS-dependent transport of carbohydrates could be correlated with acid production by metabolizing internalized carbohydrates, the glycolytic rate of the FR strain was measured using the same carbohydrates as above (Figure 7). When the reaction was initiated in the presence of glucose, sucrose, mannose, and fructose, a significant decrease in pH change of *S. mutans* wild-type and FR strains was mostly observed during the period from 5 to 10 min. While the wild-type cells rapidly lowered the pH to 4.35 ± 0.10, 4.30 ± 0.11, 4.74 ± 0.15, and 4.82 ± 0.01 after 7 min in the presence of the respective carbohydrate tested, the FR strain lowered the pH to 5.15 ± 0.24, 4.91 ± 0.08, 5.83 ± 0.07, and 5.66 ± 0.17 at the same time point. Moreover, the final pH of the wild-type strain was 3.47 ± 0.08, 3.48 ± 0.05, 3.63 ± 0.06, and 3.64 ± 0.02, respectively. In the FR strain, the final pH was 3.69 ± 0.04, 3.64 ± 0.08, 4.01 ± 0.16, and 3.80 ± 0.03, respectively. Similar to the results of the PTS assays, the FR strain produced lesser organic acids under conditions in which the limited fermentable carbohydrates were supplied than the wild-type strain (*p* < 0.05). Notably, the pH measured by the pH drop method has a strong correlation with acid tolerance [18,50]. These results are consistent with those of the stress tolerance tests, in which the FR strain showed high sensitivity to an acid stressor. Therefore, insufficient carbohydrate uptake through PTS-dependent carbohydrate transport and glycolytic pathway can partly explain the growth defects of the FR strain under stress conditions, but it did not affect the intracellular production of ATP that is important for stress response (data not shown).

## 4. Discussion

**Figure 8:**
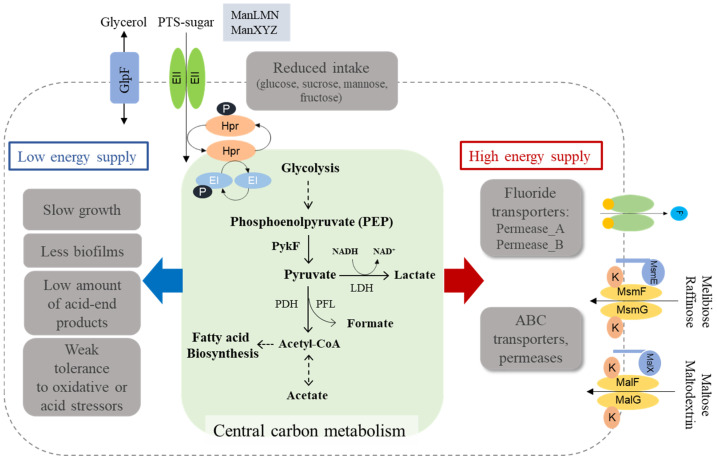
Proposed model for the mechanism of fluoride resistance in *S. mutans*. Genetic mutations in the FR genome primarily lead to the reduced activities of PEP-dependent PTS systems (e.g., ManXYZ and ManLMN), the consequence of which may strongly influence the carbon inflow and energy production via the central carbon metabolism. In contrast, the expression of putative fluoride transporters essential for fluoride resistance was highly upregulated, which suggests that more energy can be supplied for the efficient activity of these transporters. Interestingly, the upregulation of some transporters (e.g., MsmEFGK and MalXFGK) can be explained by the *S. mutans* FR cells activating the alternative transporters favorable for other substrates, to overcome the restricted carbon inflow. The red arrow indicates the upregulation of genes by genetic mutation(s) in *S. mutans* FR strain and the blue arrow indicates the downregulation and observations.

The data presented herein provide a new understanding of fluoride resistance arising from genetic mutations in the *S. mutans* genome, which are summarized in Figure 8. The concept involves the interconnection of genetic mutations conferring fluoride resistance with critical virulence attributes. Moreover, our findings reveal that the resistance mechanism integrates into a complex cellular process and carbohydrate metabolism. In particular, our results highlight the interrelationship of a reduced ability to uptake carbohydrates and a decreased flux through glycolysis, which can have an abnormal influence on the biology of *S. mutans*. Moreover, this investigation provides evidence that the accumulation of mutations to resist fluoride diminishes the expression of virulence traits such as stress tolerance and biofilm formation through, although not directly, gene expression modification.

As listed in Table 1, the FR strain isolated in the current study contained five SNPs and a multiple nucleotide insertion in the genome sequence of *S. mutans*. Genome comparisons between FR strains created by other groups revealed that the common genes (*SMU.1289c, SMU.1290c*, *glpF*, and *pykF*) containing mutations are required for fluoride resistance [12,22]. Notably, *SMU.1289c* (*permease_B, eriC1b*) and *SMU.1290c* (*permease_A, eriC1a*), known as chloride channel permeases, are major contributors to the fluoride resistance of *S. mutans*, functioning as H^+^-coupled antiporters by releasing F^-^ below the extracellular level [12,25,26]. Our results indicate that the FR strain harboring a mutation (A→G) in the intergenic region of *SMU.1289c* and *SMU.1290c* enhanced the expression level by more than fourfold (Figure 5), and this is supported by the evidence that the increased gene expression for fluoride transporters by mutations leads to the efficient export of F^-^ to the extracellular environment [51]. Furthermore, using the promoter finder (http://www.phisite.org/main/, accessed on 11 March 2021), we found one possible promoter in the intergenic region between these two genes. Consistent with the results of Liao and colleagues, mutations in the promoter of the *mutase* gene result in increased expression of the *permease_A* gene and acquired resistance [12], and a certain mutation that can modulate operon expression would provide an opportunity for acquiring fluoride resistance. Thus, the genetic variants of this operon could be considered responsible for the phenotype of fluoride resistance in cells derived from the human oral cavity [12,25,26].

While a previous study with a FR strain showed that the promoter of *glpF* gene carries a mutation in the promoter region (*glpF*p) [22], our FR strain harbored an SNP at the 464th nucleotide of a coding region that can produce a truncated GlpF protein by a nonsense mutation. A *glpF* gene encoding a glycerol uptake facilitator protein takes up glycerol into cells as a carbon source for glycolysis and as a precursor for phospholipid biogenesis [49,52]. The slow growth rate of the FR strain shown in this study may have resulted from the low expression and translation of the truncated protein [12]. However, since the experiments in this study were not performed under conditions in which glycerol was supplemented, this growth inhibition can be interpreted as a secondary effect of this mutation (Figure S2) or a combinational effect with other mutations. More interestingly, a *pykF* gene, which encodes pyruvate kinase that facilitates the conversion of PEP to pyruvate, is an important candidate gene, as shown in a previous study [12]. One possibility is that the enzymatic activity of mutated *pykF* proteins affects the conversion of PEP to pyruvate, which disturbs the sufficient transfer of phosphate groups for HPr phosphorylation, coupled with phosphoryl transfer protein enzyme I (EI). Therefore, the idea that fluoride resistance could result from the cumulative effect of multiple mutations [53] supports the notion that these common genes harboring mutations are shared in the genomes of the FR strains. 

Interestingly, we discovered that the biofilms of the FR strain were not as well established as the wild-type biofilms. This observation appeared to be correlated with the slow growth of the FR strain. In contrast, biofilm is interconnected with genetic competence [54], which suggests that biofilm of the FR strain is associated with competence phenotype. Although the transcription difference did not pass the criterion described above, the expression of the two-component system, ComDE, which involves the genetically specified ability of bacteria to uptake extracellular DNA via transformation [48,54], was significantly increased by approximately 1.5-fold. Conversely, the FR strain displayed a reduced ability to uptake the plasmid pDL278, which carries a spectinomycin resistant marker, in the presence of exogenously supplied competence-stimulating peptide (CSP), when compared to the wild-type strain (Unpublished data). Further, the verification assays with the same number of cells as the wild-type strain showed similar differences, indicating that the slow growth of the FR strain is not related to the low efficiency of transformability. However, the interrelationship between competence and fluoride resistance remains unclear. Therefore, further studies are necessary to investigate the mechanisms through which the expression of competence genes and their regulatory mechanisms are modified by mutations in the FR strain.

This study also provides insights into fluctuations in carbon flow and energy metabolism by fluoride resistance in *S. mutans*. Among the changes in overall gene expression, the expression of many of the ABC transporters (e.g., MsmEFGK and MalXFGK) and permeases (e.g., Permease_A and Permease_B) was enhanced, whereas a reduced expression for the PEP-dependent PTS systems (e.g., ManLMN and ManXYZ) was observed (Tables S2 and S3). By understanding our data along with existing knowledge, we can propose a model in which insufficient energy is allocated for persistence and virulence attributes, when the FR strain requires a high energy supply for an active transporter capable of exporting fluoride ions (Figure 8). Indeed, our results reveal that various ABC transporters and ATP-utilizing permeases, including fluoride antiporters, were upregulated, and a relative decrease in sugar uptake and glycolysis appeared in the FR strain. Although combinational effects of genetic mutations are required to elicit an effective resistance, the regulation of active membrane proteins may be seen as the most crucial basis for fluoride resistance. In addition, the downregulation of PTS-dependent sugar transport is reflected by cells restricting access to carbons, presumably leading to low energy supply to the downstream cellular processes not essential for survival. Importantly, the *mleS* gene, a malolactic enzyme for consuming malate derived from glucose, was the most downregulated in the FR strain. Malolactic fermentation stimulates the conversion of L-malate to L-lactate and CO_2_ by decarboxylation, thereby inducing the alkalization of the cytoplasm and ATP synthesis by F-ATPase [55,56,57]. This enzyme also facilitates the translocation of protons across the membrane in an ATP-dependent manner, contributing to the acid tolerance of *S. mutans* [55,56,57]. Furthermore, the expression of *gshR* (*SMU.140*), *mleP* (*SMU.138*)*,* and *oxdC* (*SMU.139*) genes, along with the *mleS* gene, is positively regulated by a regulator *mleR,* whose transcription was statistically reduced in the FR strain. The *gshR*, *mleP*, and *oxdC* genes encode putative glutathione reductase, putative malate permease, and oxalate decarboxylase, respectively. In particular, the *oxdC* gene is involved in the removal of reactive oxygen species and is co-transcribed with *mleSP* genes that contribute to the aciduricity of *S. mutans* [56]. Therefore, the downregulation of such genes partially supports the results shown in the poor stress response of the FR strain.

## 5. Conclusions

We showed that the acquisition of stable, permanent fluoride resistance through genetic mutations triggers pleiotropic effects in *S. mutans* physiology. Notably, through transcriptome analysis, the affected central carbon metabolism by insufficient intake of carbohydrates can influence the branch cellular pathways and ultimately exert aberrant consequences for the virulence and persistence of *S. mutans*. Therefore, the accumulation of genetic mutations in response to fluoride stress results in an additive effect that may reprogram complex machinery to optimize the balance between bacterial resistance and biological fitness [22,53]. Furthermore, it is necessary to consider the biological importance of these strains in addition to fluoride resistance. The daily use of fluoride-containing dental hygiene products can lead to the high survival of resistant strains in oral environments, which can be a marked advantage itself. However, considering the results shown in the current study, the possibility of resistance generation and persistence in the human oral cavity is questionable. For achieving more evidence for the above question, complementation for each mutation should be conducted to identify which genetic mutations mainly contribute to the resistant phenotype and reduced fitness in *S. mutans*. Further, the concept investigated in this study needs to be evaluated precisely in an environment that is similar to the human oral cavity in the presence of multiple species of oral microorganisms.

## Figures and Tables

**Table 1:** List of genes harboring a mutation in the FR strain.

Gene Name	Description	Mutation and Location	Effect
*hsdS*	Putative restriction endonuclease	563 A→ACAGAATTGACCT	Disruptive inframe insertion
*glpF*	Glycerol uptake facilitator protein	464 C→A	Stop codon
*pykF*	Pyruvate kinase	1156 G→A	Val→Ile
*murC2*	Putative UDP-N-acetylmuramyl tripeptide synthetase	289 G→T	Ala→Ser
*SMU.2059c*	Putative integral membrane protein	707 A→T	Tyr→Phe
*SMU.1289c* *SMU.1290c*	Chloride channel permease	A→G (Intergenic region of two genes)	

## Data Availability

All RNA-seq data were deposited in the NCBI database under accession no. GSE149509 (https://www.ncbi.nlm.nih.gov/geo/query/acc.cgi?acc=GSE149509, accessed on 11 March 2021).

## Supplementary Materials

The following are available online at https://www.mdpi.com/article/10.3390/microorganisms9040849/s1, Table S1: Oligonucleotides used in this study; Table S2: Up-regulated genes in the FR strain; Table S3: Down-regulated genes in the FR strain, Figure S1: Genetic competence; Figure S2: Growth behaviors of *S. mutans* wild-type and mutant strains obtained during growth in BHI medium.
