# Supplementary material for: Genetic Mutations That Confer Fluoride Resistance Modify Gene Expression and Virulence Traits of Streptococcus mutans
Source: Microorganisms. 2021 Apr 15;9(4):849. doi: 10.3390/microorganisms9040849 (PMC8071458; doi:10.3390/microorganisms9040849)
Supplement: Supplementary file 1 [file microorganisms-09-00849-s001.zip › microorganisms-1161557-supplementary.pdf]

Supplementary Materials

Genetic mutations that confer fluoride resistance modify gene expression and virulence traits of *Streptococcus mutans*

Hyeon-Jeong Lee[1], Jihee Song[2] and Jeong Nam Kim[1,3*]

[1] Department of Integrated Biological Science, College of Natural Sciences, Pusan National University, Busan 46241, Korea
[2] Department of Family, Youth, and Community Sciences, University of Florida, Gainesville, FL 32611, USA
[3] Department of Microbiology, College of Natural Sciences, Pusan National University, Busan 46241, Korea
* Correspondence: kimjn@pusan.ac.kr; Tel.: +82-51-510-2269

Table S1. Oligonucleotides used in this study.

Target gene	Orientation	Sequence (5' to 3')
16S rRNA	Forward	CTTACCAGGTCTTGACATCCCG
	Reverse	ACCCAACATCTCACGACACGAG
glpF	Forward	CTACTGGACCTGCCCTTAATC
	Reverse	GAATACCACCACTTTGAGTTACC
hsdS	Forward	TGGGAAGTGACAATTTGGGATA
	Reverse	CTGAGACCACCATTTGAGAGAG
pykF	Forward	GGTAGAAATCCGTGGTGGTAAA
	Reverse	ACATTGGCTCCTTCTGTAATCA
SMU.1289c	Forward	GCCCATGGCAGCTATACTATT
	Reverse	TGAGAGGTCAAACTAGCCGTA
SMU.1290c	Forward	AGGGCTGAGTACTGATTTGGTT
	Reverse	CGCCTTGATAACCTGCTGATA
murC2	Forward	CTGTCCTTCTCAATGCCAATTATG
	Reverse	CGGACACCACCTGTAAAGATT
SMU 2059c	Forward	CATCCTTACCCATTATCGTAGCA
	Reverse	AGAACCGACTCCAACCAAAG

Table S2. Up-regulated genes in the FR strain ($P < 0.01$).

Gene name	Fold change (Log2 value)	Description
SMU.1367c (*ubiE*)	6.657	conserved hypothetical protein
SMU.932	4.154	hypothetical protein
SMU.935	4.138	putative amino acid ABC transporter, permease protein
SMU.934	4.011	putative amino acid ABC transporter, permease protein
SMU.933	3.916	putative amino acid ABC transporter, periplasmic amino acid-binding protein
SMU.936	3.869	putative amino acid ABC transporter, ATP-binding protein
SMU.1341c, *SMU.1340* (*bacA2*)	2.930	putative gramicidin S synthetase / putative surfactin synthetase
SMU.1339 (*bacD*)	2.918	putative bacitracin synthetase
SMU.1342 (*bacA1*)	2.846	putative bacitracin synthetase 1
SMU.1343c	2.714	putative polyketide synthase
SMU.1568 (*malX*)	2.659	putative maltose/maltodextrin ABC transporter, sugar-binding protein
SMU.1898, *SMU.1899*	2.594	putative ABC transporter, ATP-binding and permease protein
SMU.1345c	2.455	putative peptide synthetase similar to MycA
SMU.503c	2.353	hypothetical protein
SMU.651c	2.351	putative ABC transporter, substrate-binding protein
SMU.1344c	2.302	putative malonyl-CoA-acyl-carrier-protein transacylase
SMU.1289c (*permease_B*, *eriC1b*)	2.299	putative permease, chloride channel
SMU.1336 (*pksD*)	2.261	conserved hypothetical protein, involved in polyketide synthesis
SMU.962	2.260	putative dehydrogenase
SMU.961	2.232	conserved hypothetical protein
SMU.609	2.199	putative 40K cell wall protein precursor
SMU.1564 (*glgP*), *smu.1565* (*malQ*)	2.147	putative glycogen phosphorylase / putative 4-alpha-glucanotransferase
SMU.1293c	2.134	conserved hypothetical protein
SMU.1290c (*permease_A*, *eriC1a*), *SMU.1291c* (*mut*)	1.984	putative permease, chloride channel / putative chorismate mutase
SMU.1292c	1.967	conserved hypothetical protein
SMU.1184c	1.939	putative transcriptional regulator, antiterminator

SMU.652c, *SMU.653c*	1.930	putative ABC transporter, ATP-binding protein, possible nitrate transport system / putative ABC transporter, permease protein
SMU.1569 (*malF*), *SMU.1570* (*malG*)	1.927	putative maltose/maltodextrin ABC transporter, permease protein
SMU.1335c	1.926	putative enoyl-(acyl-carrier-protein) reductase
SMU.1575c, *SMU.1576c*	1.923	hypothetical protein
SMU.1183 (*mtlA2*)	1.900	PTS system, mannitol-specific enzyme IIA
SMU.1157c	1.865	conserved hypothetical protein
SMU.984	1.827	hypothetical protein
SMU.1337c	1.820	putative alpha/beta superfamily hydrolase
SMU.1347c, *SMU.1346* (*bacT*)	1.800	conserved hypothetical protein, possible permease / putative thioesterase
SMU.840c	1.789	hypothetical protein
SMU.1160c	1.725	hypothetical protein
SMU.877 (*agaL*)	1.720	alpha-galactosidase
SMU.43	1.715	putative site-specific DNA-methyltransferase restriction-modification protein
SMU.1650 (*end3*)	1.704	putative endonuclease III (DNA repair)
SMU.1365c	1.685	hypothetical protein, possible permease
SMU.1048	1.680	conserved hypothetical protein
SMU.930c	1.661	putative transcriptional regulator
SMU.1287	1.645	putative transcriptional regulator
SMU.1295 (*add*)	1.645	putative adenosine deaminase
SMU.1182 (*mtlD*)	1.610	mannitol-1-phosphate dehydrogenase
SMU.1571	1.586	putative ABC transporter, ATP-binding protein
SMU.1577c	1.579	conserved hypothetical protein
SMU.1334 (*sfp*)	1.569	putative phosphopantetheinyl transferase
SMU.878 (*msmE*)	1.566	multiple sugar-binding ABC transporter, sugar-binding protein precursor
SMU.605	1.564	hypothetical protein
SMU.1771c, *SMU.1772c*, *SMU.1773c*	1.519	hypothetical protein
SMU.895, *SMU.896*	1.505	possible DNA-damage-inducible protein / conserved hypothetical protein
SMU.44	1.490	possible DNA mismatch repair protein
SMU.431	1.450	putative ABC transporter, ATP-binding protein
SMU.1069c, *SMU.1070c*	1.445	hypothetical protein / conserved hypothecial protein

SMU.879 (*msmF*)	1.429	multiple sugar-binding ABC transporter, permease protein
SMU.684,<br>*SMU.685*	1.423	hypothetical protein
SMU.1338c	1.403	putative permease, possible multidrug-efflux transporter
SMU.1067c,<br>*SMU.1068c*	1.380	putative ABC transporter, permease protein / putative ABC transporter, ATP-binding protein
SMU.432	1.372	putative ABC transporter, integral membrane protein
SMU.985 (*bglA*)	1.368	putative beta-glucosidase
SMU.45	1.351	hypothetical protein
SMU.986c	1.350	hypothetical protein
SMU.880 (*msmG*)	1.335	multiple sugar-binding ABC transporter, permease protein
SMU.510c	1.335	hypothetical protein
SMU.876 (*msmR*)	1.327	putative MSM operon regulatory protein
SMU.1185 (*mtlA1*)	1.322	PTS system, mannitol-specific enzyme IIBC component
SMU.1294 (*flaW*)	1.317	putative flabodoxin
SMU.1288 (*rl19*)	1.306	50S ribosomal protein L19
SMU.1052	1.293	conserved hypothetical protein
SMU.1574c	1.218	conserved hypothetical protein
SMU.560c	1.204	conserved hypothetical protein
SMU.897	1.200	putative type I restriction-modification system, helicase subunit
SMU.998	1.200	putative ABC transporter, periplasmic ferrichrome-binding protein
SMU.2027	1.199	putative transcriptional regulator
SMU.1000,<br>*SMU.999*	1.191	hypothetical protein
SMU.561c	1.183	putative hydrolase
SMU.136c	1.183	putative transcriptional regulator
SMU.1366c	1.177	putative ABC transporter, ATP-binding protein
SMU.1126 (*coaA*)	1.175	putative pantothenate kinase
SMU.46,<br>*SMU.47*	1.172	hypothetical protein
SMU.1161c	1.171	hypothetical protein
SMU.1348c	1.171	putative ABC transporter, ATP-binding protein
SMU.2165	1.164	putative SpoJ
SMU.704c	1.154	putative autolysin, amidase
SMU.1402c	1.150	conserved hypothetical protein
SMU.1404c,	1.138	conserved hypothetical protein

SMU.1405c		
SMU.2047 (*ptsG*)	1.129	putative PTS system, glucose-specific IIABC component
SMU.1037c, *SMU.1038c*	1.129	putative histidine kinase / putative response regulator
SMU.2046c	1.128	conserved hypothetical protein
SMU.606	1.126	hypothetical protein
SMU.287	1.100	putative ComB
SMU.1942c	1.092	putative amino acid binding protein
SMU.1761c, *SMU.1762c*, *SMU.1763c*	1.079	conserved hypothetical protein
SMU.1442c	1.056	conserved hypothetical protein
SMU.1941 (*atmB*)	1.048	putative membrane lipoprotein
SMU.768c	1.046	conserved hypothetical protein
SMU.881 (*gtfA*)	1.044	sucrose phosphorylase
SMU.2153c	1.034	putative peptidase
SMU.1107c	1.024	conserved hypothetical protein
SMU.804	1.015	hypothetical protein
SMU.1125c	1.011	conserved hypothetical protein
SMU.1116c	1.006	hypothetical protein
SMU.1764c	1.002	conserved hypothetical protein
SMU.682	1.000	hypothetical protein

Table S3. Down-regulated genes in the FR strain ($P < 0.01$).

Gene name	Fold change (Log2 value)	Description
SMU.182 (*sloA*), *SMU.183* (*sloB*)	-1.078	putative ABC transporter, ATP-binding protein / putative Mn/Zn ABC transporter
SMU.564	-1.151	conserved hypothetical protein
SMU.423	-1.288	hypothetical protein
SMU.1774c	-1.296	conserved hypothetical protein
SMU.278	-1.321	hypothetical protein
SMU.1877 (*ptnA*, *manL*)	-1.399	putative PTS system, mannose-specific component IIAB
SMU.1967 (*ssb2*)	-1.409	putative single-stranded DNA-binding protein
SMU.1879 (*manN*)	-1.427	putative PTS system, mannose-specific component IID
SMU.1878 (*ptnC*, *manM*)	-1.445	putative PTS system, mannose-specific component IIC
SMU.1397c	-1.593	conserved hypothetical protein
SMU.870, *SMU.872*, *SMU.871* (*pfkB*)	-1.724	putative transcriptional regulator of sugar metabolism / putative PTS system, fructose-specific enzyme IIABC component / putative fructose-1-phosphate kinase
SMU.2146c	-1.777	hypothetical protein
SMU.1960c	-1.839	putative PTS system, mannose-specific IIB component
SMU.1961c (*manX*)	-1.880	putative PTS system, sugar-specific enzyme IIA component
SMU.1958c (*manY*)	-2.000	putative PTS system, mannose-specific IIC component
SMU.1956c	-2.115	hypothetical protein
SMU.1957 (*manZ*)	-2.182	putative PTS system, mannose-specific IID component
SMU.141	-3.567	conserved hypothetical protein
SMU.140	-3.779	putative glutathion reductase
SMU.138	-3.797	putative malate permease
SMU.139	-3.896	conserved hypothetical protein
SMU.137 (*mleS*)	-4.084	malolactic enzyme

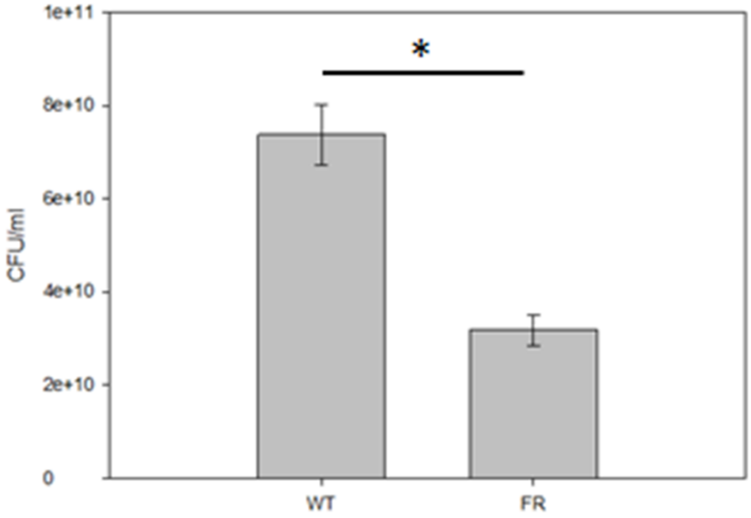

Figure S1. CFU measurement of *S. mutans* wild-type and FR strains. Early exponential cultures grown in BHI medium were diluted 1:100 in a semi-defined biofilm medium (BM). Following 24 h of incubation at 37 °C in a 5% CO_2 aerobic atmosphere, serial dilutions of transformants were plated on BHI medium to determine colony forming units (CFUs). The results are expressed as means of triplicates of three independent isolates. *****, differs from the wild-type strain at $P < 0.05$.

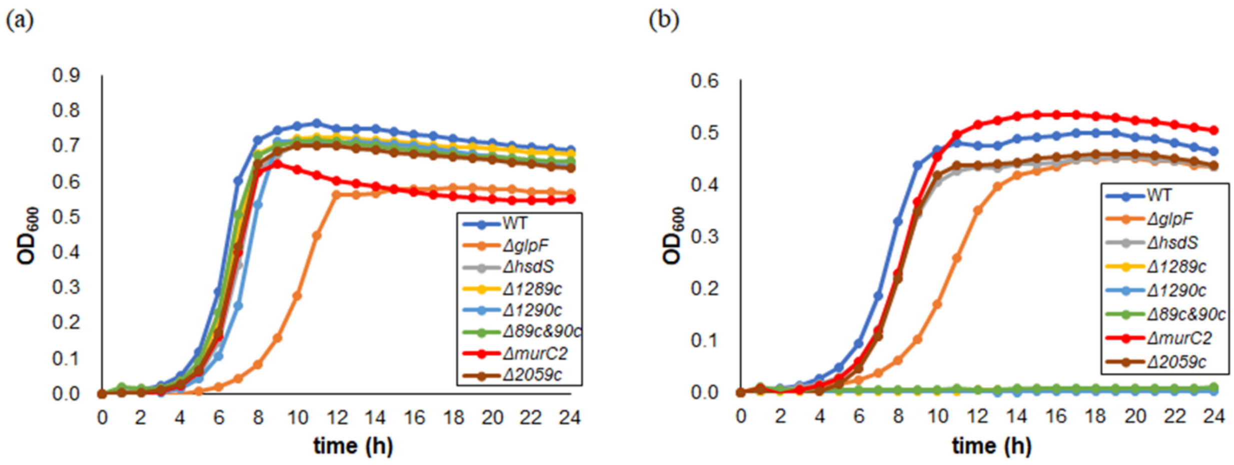

Figure S2. Growth behaviors of *S. mutans* wild-type and mutant strains obtained during growth in BHI medium. The eight strains, wild-type, *ΔglpF*, *ΔhsdS*, *ΔSMU.1289c*, *ΔSMU.1290c*, *ΔSMU.1289c&1290c*, *ΔmurC2* and *ΔSMU.2059c* were grown at 37 °C (a) without NaF under 5% CO_2 conditions or (b) with 100 μg mL^{-1} NaF. Optical density at 600 nm (OD_{600}) was monitored every 1 h at 37 °C using the Bioscreen C Lab System. The results are expressed as means of triplicates of three independent isolates.
